# Rapid, Abiotic Nitrous Oxide Production from Fe(II)-Driven Nitrate Reduction Governed by pH Under Acidic Conditions

**DOI:** 10.3390/molecules30234580

**Published:** 2025-11-28

**Authors:** Liping Xu, Fei Ma, Jianmin Zhou, Changwen Du

**Affiliations:** 1 The State Key Laboratory of Soil and Sustainable Agriculture, Institute of Soil Science, Chinese Academy of Sciences, Nanjing 210008, China; xuliping@issas.ac.cn (L.X.); fma@issas.ac.cn (F.M.); jmzhou@issas.ac.cn (J.Z.); 2 College of Advanced Agricultural Sciences, University of Chinese Academy of Sciences, Beijing 100049, China

**Keywords:** ferrous iron, nitrate reduction, nitrous oxide, Fourier-transform infrared attenuated total reflectance

## Abstract

Denitrification is conventionally viewed as a microbially mediated process driven by organic carbon, but the coupling of iron and nitrogen cycles represents a significant alternative pathway. Previous research focused on alkaline environments, leaving the direct reaction between ferrous iron (Fe^2+^) and nitrate (NO_3_^−^) in acidic conditions poorly understood. This study investigated this process using in situ spectroscopy, examining the effects of the reactant ratio, time, temperature, and initial pH while monitoring nitrous oxide (N_2_O) production. Results showed that the reaction was rapid, with most nitrate reduction within 5 min. The Fe^2+^ to NO_3_^−^ molar ratio had a limited influence on efficiency. Temperature had a non-monotonic effect, optimal at 25 °C. The initial pH was the dominant control, with lower pH (e.g., 4.6) essential for high efficiency. Crucially, the process was confirmed as a significant source of N_2_O under anoxic conditions. This work confirms Fe^2+^-driven nitrate reduction is a fast, acid-dependent process governed by pH and modulated by temperature. These findings revise our understanding of nitrogen fate and N_2_O emissions and warn of potential underestimation of nitrate in samples containing Fe^2+^.

## 1. Introduction

Denitrification is a critical microbial process in the biogeochemical nitrogen cycle, serving as a major sink for fixed nitrogen by converting reactive species back into gaseous forms returned to the atmosphere [1,2]. This process mitigates the accumulation of excess reactive nitrogen in terrestrial ecosystems, helping to prevent soil acidification and nitrate leaching. However, it also exerts a dual influence on global environmental change through the emission of gaseous intermediates, notably the potent greenhouse gas nitrous oxide (N_2_O), which contributes to climate forcing and stratospheric ozone depletion [3,4,5,6]. Conventional soil science has long framed heterotrophic denitrification within a paradigm where organic carbon serves as the exclusive electron donor, effectively isolating the nitrogen cycle. This view is now being revised by compelling evidence for a robust coupling between the iron and nitrogen cycles, specifically through the direct or indirect participation of Fe(II) and Fe(III) as electron donor and acceptor in nitrogen transformations. This interaction is now recognized as a critical control point for soil nitrogen retention and loss [7,8].

Iron, the fourth most abundant element in the Earth’s crust, is indispensable for biological metabolism. Its profound biogeochemical influence stems from its redox versatility, readily cycling between ferrous (Fe^2+^) and ferric (Fe^3+^) states, which function as a potent electron donor and acceptor, respectively [9,10]. Consequently, iron-mediated denitrification pathways have been extensively documented across diverse anaerobic environments, including paddy soils, submarine sediments, surface waters, and activated sludge systems [11,12,13,14,15]. Current literature synthesizes iron-dependent denitrification into biotic, abiotic, and coupled biotic-abiotic pathways. A foundational biotic mechanism involves Dissimilatory Iron-Reducing Bacteria (DIRB), which couple the oxidation of organic carbon to the reduction of Fe^3+^ (oxy)hydroxides [16,17]. The resulting Fe^2+^ can subsequently be oxidized by chemolithoautotrophic microorganisms that reduce nitrate under anaerobic conditions, a process termed Nitrate-Reducing Fe^2+^ Oxidation (NRFO) [18,19]. This reaction is stoichiometrically represented as

10Fe^2+^ + 2NO_3_^−^ + 24H_2_O → 10Fe(OH)_3_ + N_2_ + 18H^+^ (1)

Significant potential also exists for abiotic chemical pathways. Field observations of co-varying gradients between nitrate and extractable Fe^2+^ at oxic-anoxic interfaces have long supported the hypothesis that Fe^2+^ acts as a direct chemical reductant for nitrate (NO_3_^−^) [18,20], further corroborated by the rapid oxidation of native soil Fe^2+^ upon nitrate introduction [21]. Prevailing explanations often attribute such abiotic nitrate reduction to reactions between nitrite and Fe^2+^ [22,23,24] or to electron transfer from Fe^2+^-bearing minerals like green rust [25,26]. A pivotal, yet often implicit, assumption underpinning these conclusions is that the reaction between Fe^2+^ and NO_3_^−^ requires catalysts—such as electrocatalysis, metal ions, or metal compounds [27,28]—and that in the absence of catalytic surfaces or nitrite intermediates, aqueous Fe^2+^ cannot directly reduce nitrate. A critical re-examination of foundational studies, however, reveals nuances that challenge this assumption. Early works by Hansen et al. concluded that no significant reaction occurred at pH ≈ 8.25 based on minimal ammonium production [29,30]. Similarly, Ottley et al., working at pH 8.0, reported slow abiotic nitrate reduction (~15% over one week) [31]. It is noteworthy that the extended observation intervals in these studies may have failed to capture faster initial reaction kinetics. Crucially, their primary focus—along with other seminal studies [32,33,34]—was on alkaline conditions and the pathway of nitrate reduction to ammonium (NH_4_^+^), leaving the kinetics and products of the direct Fe^2+^-NO_3_^−^ interaction in acidic systems comparatively unexplored.

Emerging evidence from acidic environments challenges this alkaline-centric view. Xu and Cai demonstrated a widespread coupling between Fe^2+^ oxidation and NO_3_^−^ reduction in anaerobic, acidic subtropical soils (pH (H_2_O) < 5) [22]. Critically, research by Wang et al. indicates that nitrate-dependent Fe(II) oxidation can be a significant source of N_2_O in paddy soils, with emission magnitudes regulated by environmental Fe^2+^ levels [35]. The electron transfer stoichiometry can be conceptualized as

Fe^2+^ − e^−^ → Fe^3+^ (2)

NO_3_^−^ + 2e^−^ + 2H^+^ → NO_2_^−^ + H_2_O (3)

NO_2_^−^ + 2e^−^ + 3H^+^ → 0.5N_2_O + 1.5H_2_O (4)

0.5N_2_O + e^−^ + H^+^ → 0.5N_2_ + 0.5H_2_O (5)

Supporting this, a suspension culture study by Petersen et al. demonstrated that the transformation pathway likely involves both biotic and abiotic reactions, with N_2_O being a prominent gaseous product [12]. Furthermore, Fu et al. reported that at pH 6.20 and in the absence of biological activity, nitrate-nitrogen reduction by zero-valent iron (a persistent source of Fe^2+^) reached 18.3% via purely chemical reactions [27]. These findings coalesce into a critical, unresolved question: Does significant abiotic Fe^2+^-mediated nitrate reduction occur under moderately acidic conditions, and if so, is N_2_O a key product? Resolving this question is complicated by profound analytical challenges in nitrate quantification. Conventional methods—including phenol disulfonic acid spectrophotometry, ion chromatography, cadmium column reduction, and ultraviolet spectrophotometry—are all susceptible to interference from soluble iron or its precipitates [36,37,38]. For instance, flow injection analysis with a standard NH_4_Cl–EDTA buffer systematically underestimates NO_3_^−^ in the presence of soluble iron [39]. While alternatives like an imidazole buffer exist, they risk analytical artifacts from metal hydroxide precipitation [40]. Ultraviolet spectrophotometry is compromised by the intrinsic absorption of iron ions, leading to substantial overestimation [41]. Even ion chromatography requires rigorous pre-treatment to prevent system degradation from iron oxidation.

Given these constraints, mid-infrared spectroscopy has emerged as a promising alternative. Fourier-Transform Infrared Attenuated Total Reflectance (FTIR-ATR) spectroscopy has been successfully applied to nitrate determination with minimal interference [42,43]. Shao et al. utilized this method to analyze nitrate in aqueous solutions and soils, identifying a characteristic absorption peak in the 1200–1500 cm^−1^ region [44]. Pertinently, they raised the possibility that measurement discrepancies in flooded soils could stem from a reaction between nitrate and Fe^2+^ during the extraction process itself [44]. Recently, Xu et al. demonstrated that FTIR-ATR coupled with partial least squares regression achieves reliable quantification of NO_3_^−^ across a wide concentration range in water (R^2^ = 0.99) without extensive pretreatment [45].

Therefore, this study employs FTIR-ATR spectroscopy to investigate the direct reaction between Fe^2+^ and NO_3_^−^ in a well-defined aqueous system under varying substrate concentrations, stoichiometric ratios, temperatures, and initial pH levels. Concurrently, photoacoustic spectroscopy is utilized to monitor N_2_O production. The objective is to unequivocally determine the occurrence, kinetics, gaseous product profile, and key controlling factors of this iron-mediated denitrification pathway under moderately acidic conditions.

## 2. Results

### 2.1. Influence of Mass Ratio on Nitrate Reduction Efficiency

**Figure 1:**
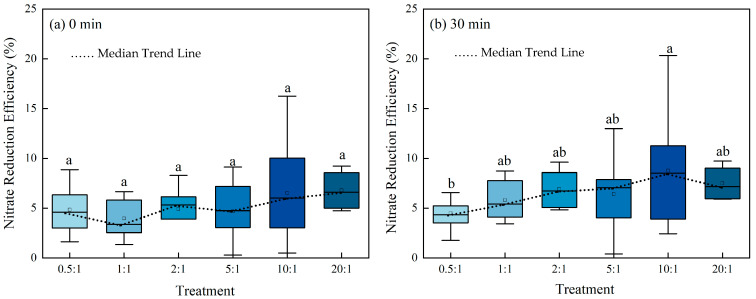
Nitrate reduction efficiency under different mass ratios. Data represent the nitrate reduction efficiencies across all treatments at 0 min (**a**) and 30 min (**b**) of reaction time, respectively. The varying shades of blue are used for visual clarity and have no quantitative significance. Lowercase letters above the bars indicate significant differences between Fe^2+^:NO_3_^−^ molar ratios (0.5:1, 1:1, 2:1, 5:1, 10:1, and 20:1) at the same time point (*p* < 0.05, one-way ANOVA with Duncan’s test). Bars not sharing a common letter are significantly different. The black dotted lines represent the median nitrate reduction efficiencies, respectively.

Evaluation of nitrate reduction efficiency across a range of mass ratios (0.5:1 to 20:1) revealed distinct trends (Figure 1). The clear separation between the median trend lines at 0 min and 30 min indicates that nitrate reduction is associated with reaction time. To further investigate the role of the mass ratio, a comparative analysis across the different ratios was conducted. For the 30 min data, a slight upward trend in median values was observed with increasing mass ratio. However, the incremental gains between adjacent ratios were often comparable to the 3% measurement error, suggesting that any positive correlation is of secondary practical importance compared to the dominant time effect. The substantial data dispersion at 30 min indicates genuine process-related variability and confirms that mass ratio alone is not the sole performance determinant. While mass ratio and time are foundational parameters, achieving higher and more consistent reduction efficiency likely depends on optimizing other key variables. These findings establish a basis for investigating critical operational parameters such as pH and temperature to fine-tune the reduction process.

### 2.2. Rapid Nitrate Reduction

Based on preliminary screening, molar ratios of n(Fe^2+^):n(NO_3_^−^) = 5:1 and 10:1 were selected for detailed kinetic study. These ratios bracket the effective range where noticeable enhancement in nitrate reduction was observed, avoiding regions of diminishing returns or high data variability.

**Figure 2:**
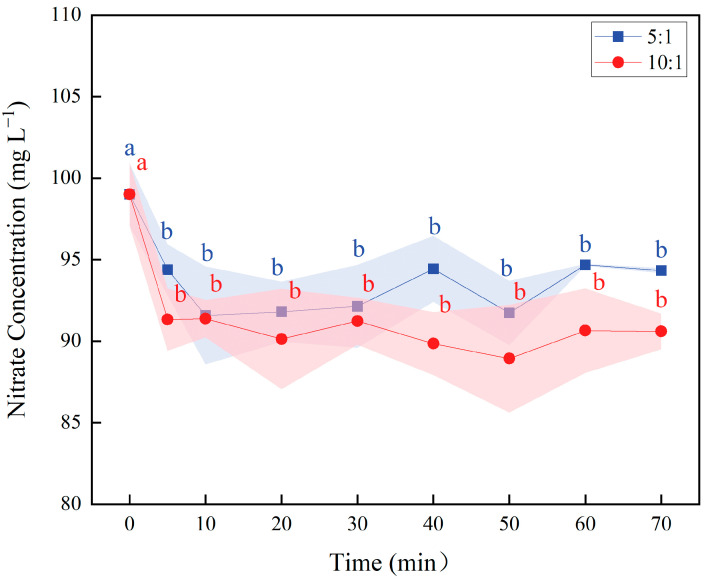
Temporal changes in nitrate concentration under different mass ratios. Note: Shaded area represents the mean ± standard deviation (SD) (*n* = 3). Blue and red lowercase letters above data points indicate statistically significant differences among different time points (*p* < 0.05, one-way ANOVA with Duncan’s test) for Fe^2+^:NO_3_^−^ molar ratios of 5:1 and 10:1. Data points not sharing a common letter are significantly different.

The time-course study revealed a rapid initial phase, with a significant decrease in nitrate concentration occurring within the first 5 min, followed by a plateau where no further significant reduction was observed (Figure 2). This indicates that the iron-mediated nitrate reduction proceeds rapidly at room temperature, with the reaction largely completing within min.

### 2.3. Influence of Reaction Temperature

**Figure 3:**
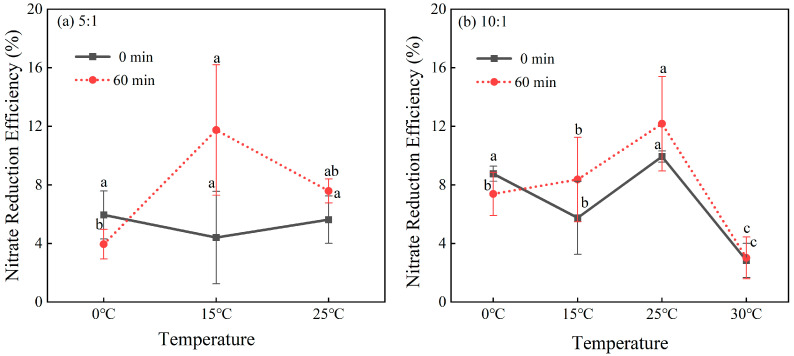
Nitrate reduction efficiency under different temperatures. Data are shown for Fe^2+^:NO_3_^−^ molar ratios of 5:1 (**a**) and 10:1 (**b**). The solid and dotted lines represent the data trends at different reaction time points (0 min and 30 min, respectively). Error bars represent the standard deviation of replicate experiments. Different lowercase letters indicate statistically significant differences among the tested conditions (*p* < 0.05, one-way ANOVA with Duncan’s test). Data points not sharing a common letter are significantly different.

Nitrate reduction efficiency was investigated as a function of temperature across different Fe^2+^:NO_3_^−^ molar ratios and reaction times (Figure 3). The data demonstrate that reaction temperature has a substantial and non-monotonic impact on process efficiency. Nitrate reduction was completely suppressed at 0 °C, with efficiencies remaining near baseline levels, underscoring the reaction’s critical dependence on thermal energy for initiation. A pronounced and statistically significant enhancement occurred at 25 °C, representing peak performance under the tested conditions. However, a further increase to 30 °C resulted in a clear decrease in efficiency, indicating an upper operational limit.

The robustness of this temperature-dependent relationship—characterized by activation, optimization at 25 °C, and subsequent decline—is demonstrated by its consistent manifestation across both the 5:1 and 10:1 molar ratios. These results confirm that the reaction occurs significantly across a range of common environmental temperatures, with its efficiency being substantially governed by thermal conditions. Identification of this distinct temperature profile is essential for a mechanistic understanding of the underlying reaction.

### 2.4. Role of Initial pH in the Nitrate Reduction

**Figure 4:**
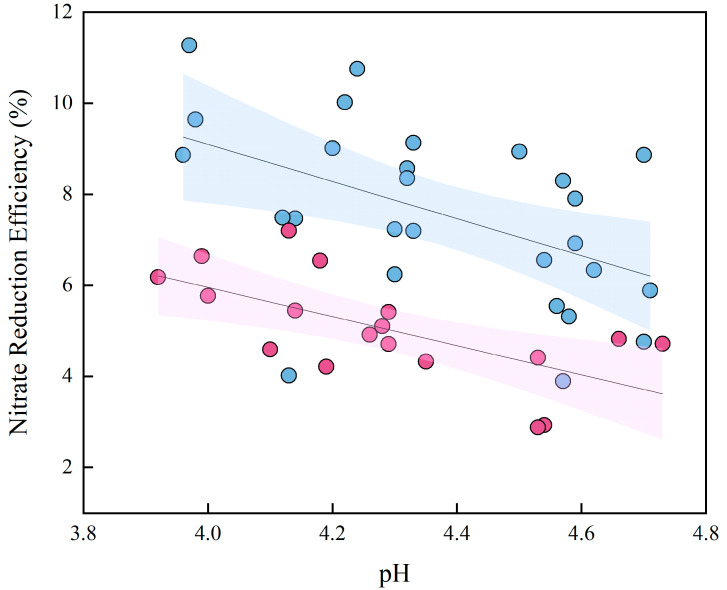
Scatter plot of solution pH versus nitrate reduction efficiency at different initial pH levels. Scatter plots are categorized by the initial pH of the solution before Fe^2+^ addition, with data for pH 5.6 and 6.6 shown in blue and purple circles, respectively. The depicted regression lines and their accompanying shaded areas represent the best-fit models and the 95% confidence intervals, respectively.

Initial experiments, conducted with distilled water equilibrated with atmospheric CO_2_, incidentally produced data under two approximate initial pH conditions. Analysis of this data revealed a clear negative correlation, where the nitrate reduction efficiency decreased from 10–12% to merely 2–4% as the solution pH increased from approximately 3.8 to 4.8 (Figure 4). This pronounced inverse relationship indicates that a more acidic environment is fundamentally more favorable for nitrate reduction in this system. The data segregate into two distinct clusters based on initial pH. Systems initiated at the lower pH of 5.7 demonstrated superior performance, achieving the highest nitrate reduction efficiencies (up to 12%) at the lower end of the solution pH spectrum (3.8–4.2). In contrast, systems starting at the higher initial pH of 6.6 were predominantly clustered in the region of higher solution pH (above 4.4) and lower reduction efficiency (mostly below 6%). This pattern unequivocally shows that an initial pH of 5.7 is more effective, facilitating both higher maximum reduction rates and greater efficiency across the observed pH range.

**Figure 5:**
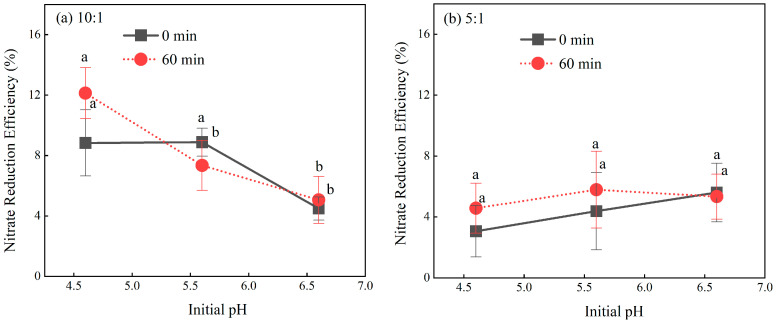
Nitrate reduction efficiency under different initial pH conditions. Data points are distinguished by initial pH values (4.6, 5.6 and 6.6) and substrate ratios of 5:1 (**a**) and 10:1 (**b**), indicated by different symbols). The solid and dotted lines represent the data trends at different reaction time points (0 min and 30 min, respectively). Letters above data distributions indicate significant differences (*p* < 0.05) between initial pH groups based on one-way ANOVA with Duncan’s test. Data points not sharing a common letter are significantly different.

Building on these findings, the experimental matrix was expanded to include three controlled initial pH levels (4.6, 5.6 and 6.6) and two molar ratios (5:1 and 10:1). The analysis confirms a robust negative correlation between final solution pH and nitrate reduction efficiency across all conditions (Figure 5). Data points corresponding to the lowest initial pH of 4.6 consistently achieved the highest nitrate reduction efficiencies, predominantly clustering in the region of high reduction efficiency (>8%) and lower final solution pH (<4.0). Within this framework, the 10:1 molar ratio demonstrated generally superior nitrate reduction efficiency compared to the 5:1 ratio across the pH spectrum. For instance, at comparable final pH values around 4.0, systems with the 10:1 ratio typically showed reduction rates 2–4 percentage points higher than those with the 5:1 ratio. This effect was most pronounced when combined with lower initial pH conditions. However, the data clearly demonstrate that initial pH exerts the predominant influence on system performance. A higher initial pH (e.g., 6.6) results in a final pH that remains in a less optimal range (>4.5), correlating with diminished reduction efficiency regardless of the substrate ratio.

In conclusion, while substrate availability provides secondary modulation, the initial pH emerges as the predominant and decisive factor controlling nitrate reduction. The experimental evidence establishes that a lower initial pH is the primary prerequisite for creating the persistently acidic environment required to maximize the nitrate reduction process.

### 2.5. N_2_O Production Dynamics Under Varied Experimental Conditions

**Figure 6:**
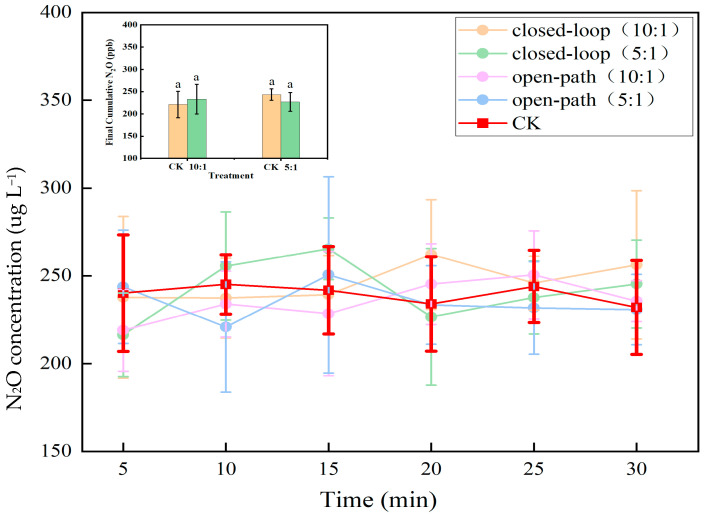
N_2_O production dynamics and final yield against an ambient air background. Data were averaged and plotted at 5 min intervals (*n* = 5). The closed-loop technique, where the instrument’s inlet and outlet formed a closed circuit with the reaction vessel for repeated headspace analysis, and the open-path technique, where the reaction vessel remained open to allow for real-time measurement with atmospheric exchange. The inset bar chart highlights the cumulative N_2_O production at the 60 min endpoint, with different lowercase letters denoting statistically significant differences between CK and treatments (*p* < 0.05).

Initial measurements conducted against an ambient air background revealed no significant N_2_O production. As shown in Figure 6, the N_2_O concentration trajectories for both closed-loop and open-path techniques showed no statistically significant deviation from the control over 30 min. All three groups exhibited minor fluctuations around a similar baseline, with no consistent increasing trend. The substantial overlap between the error bars of the treatment groups and the control at every time point indicates that within-group variability was greater than any observable difference between groups. This lack of a clear signal was corroborated by the 60 min cumulative production data (Figure 6 inset), where any potential treatment effect was masked by the substantial and variable ambient N_2_O background.

**Figure 7:**
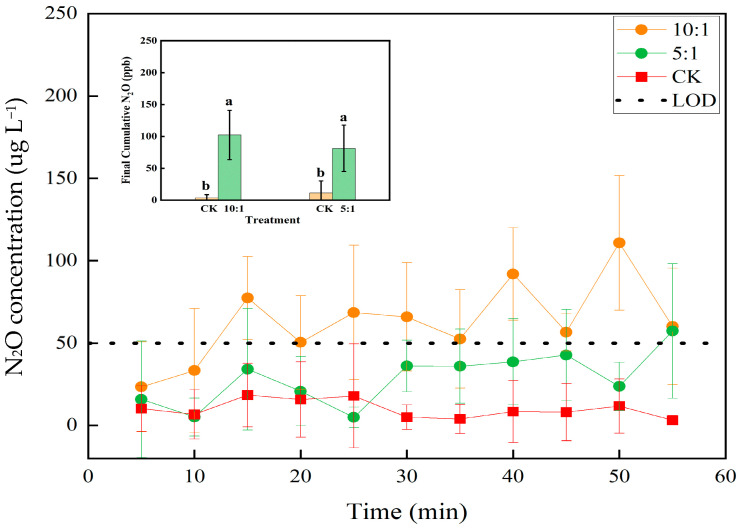
Fe^2+^-Driven Nitrate Reduction Significantly Enhances Cumulative N_2_O Production under N_2_ Atmosphere. Values are means ± SD (*n* = 3) calculated at 5 min intervals. The dashed line corresponds to the limit of detection (LOD = 50 ug L^−1^) for N_2_O. The inset bar chart highlights the cumulative N_2_O production at the 60 min endpoint, with different lowercase letters denoting statistically significant differences between CK and treatments (*p* < 0.05).

To conclusively determine the potential for N_2_O production, the experiment was repeated under a controlled anoxic (N_2_) background. This yielded a markedly different outcome (Figure 7). Both the 10:1 and 5:1 Fe^2+^:NO_3_^−^ molar ratios induced a clear and steady increase in N_2_O, with concentrations decisively diverging from the stable control baseline after 20–30 min. The control values remained consistently near baseline in the pure N_2_ environment. The statistical significance of this production was confirmed by analysis of the final cumulative yields (Figure 7 inset). Pairwise comparisons affirmed that both the 10:1 and 5:1 treatments resulted in a statistically significant increase in N_2_O accumulation compared to the control.

Collectively, the results from the anoxic system provide conclusive evidence that the abiotic reduction of nitrate by Fe^2+^ is a significant source of N_2_O, a signal that was only distinguishable from background variability under a purified N_2_ atmosphere.

## 3. Discussion

Previous studies have concluded that the reaction between Fe^2+^ and NO_3_^−^, while thermodynamically feasible, proceeds slowly without a catalyst [29,30,31]. This established view, however, contrasts with the rapid reaction kinetics observed in our aqueous system. The discrepancy can be attributed to two key factors. First, the aforementioned studies were conducted under alkaline conditions, whereas our work demonstrates that moderately acidic conditions are markedly more favorable for the reaction in solution. Second, the relatively high reactant concentrations used in our system likely increased the number of activated molecules and the frequency of effective collisions, thereby accelerating the reaction rate.

Regarding the underlying mechanism, it remains challenging to definitively conclude whether the process is purely abiotic. The distilled water used in our experiments was not sterilized, leaving open the possibility of microbial involvement. This methodological consideration is supported by the control experiments of Ottley et al., who observed negligible nitrate reduction within 24 h but approximately 15% reduction over one week under supposedly catalyst-free conditions [31]. The authors explicitly acknowledged that complete sterilization could not be guaranteed, suggesting that slow, microbially mediated reduction could not be ruled out. This provides an important context for interpreting our own results, where rapid kinetics suggest a dominant abiotic pathway, though a minor biotic contribution cannot be entirely discounted. To unequivocally confirm the abiotic nature of the reaction in future work, it is critical to employ rigorous sterilization techniques for the experimental water. A definitive approach would be to apply the moist heat sterilization method mandated by pharmacopoeial standards (e.g., autoclaving at 121 °C for 15–30 min) [46]. For a more practical laboratory-scale solution, pasteurization (circulating water at 80 °C ± 5 °C for 1–2 h), which is recommended for controlling microbial growth in water systems, can be utilized. Either method would serve to eliminate potential microbial contributors to the reaction.

**Figure 8:**
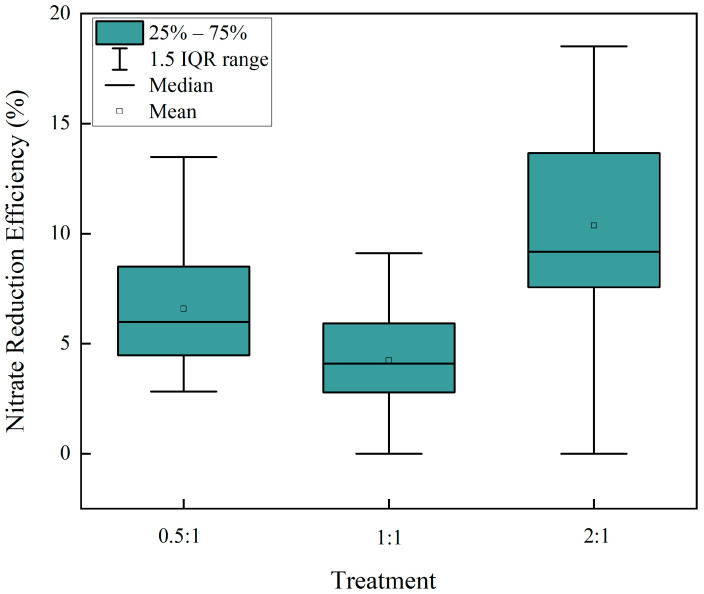
Nitrate reduction efficiency under different mass ratios. Data are shown for Fe^2+^:NO_3_^−^ molar ratios of 0.5:1; 1:1 and 2:1. Error bars represent the standard deviation of replicate experiments.

Classical kinetics would predict that an increase in temperature enhances reaction rates by providing molecules with sufficient energy to overcome the activation barrier. Contrary to this expectation, we observed a suppression of nitrate reduction when the temperature was raised to a constant 30 °C. A plausible explanation is that the elevated temperature increased the concentration of dissolved oxygen, thereby accelerating the competitive oxidation of Fe^2+^ [47]. This side reaction would consume the reductant and prematurely terminate the target nitrate reduction. The overall low nitrate reduction efficiency (~10%) observed under ambient conditions may also be linked to this oxidative competition. To test this hypothesis, we conducted experiments under anaerobic conditions (N_2_-purged) for substrate ratios that exhibited lower reduction rates. The results (Figure 8) confirm a significant enhancement in nitrate reduction efficiency in the absence of O_2_, providing direct evidence supporting our conjecture.

**Figure 9:**
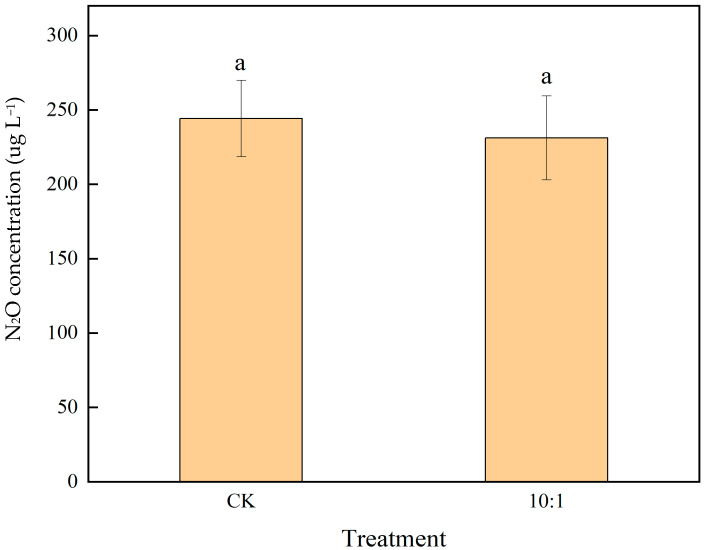
N_2_O final yield against an ambient air background. Error bars represent the standard deviation of replicate experiments. Letters denoting statistically significant differences between CK and treatments (*p* < 0.05, one-way ANOVA with Duncan’s test). Bars sharing a common letter are not significantly different.

The selection of a 100 mg L^−1^ nitrate concentration for this study was guided by methodological and practical considerations. This concentration falls within the 10–100 mg L^−1^ range where the analytical error of our FTIR-ATR method is minimized (<3 mg L^−1^) [45]. Furthermore, this level is environmentally relevant for natural water and soil systems while providing a sufficient magnitude of chemical change to reliably monitor reaction progress in a laboratory setting. Concerning reaction products, the quantities generated under ambient air conditions were insufficient to be distinguished from the variable atmospheric N_2_O background. Even when the reactant concentration was increased to 500 mg L^−1^ (as N), no significant product signal was detected after 30 min of reaction (Figure 9). In contrast, conclusive evidence identifying N_2_O as a reaction product was successfully obtained under an N_2_ background. While the anaerobic condition alters the system’s redox state, these results allow us to preliminarily conclude that the reaction between Fe^2+^ and NO_3_^−^ can proceed abiotically in aqueous solution, with N_2_O as a key gaseous product.

While NO_3_^−^ and N_2_O were unequivocally identified in this study, the complete fate of nitrogen—such as the potential formation of N_2_ or NH_4_^+^—was not fully constrained. To establish a comprehensive nitrogen mass balance in future work, it will be essential to quantify NH_4_^+^ concentrations and other gaseous nitrogen species. Isotopic analysis offers a powerful approach for tracing nitrogen transformations. Enzymatic nitrate reduction typically enriches the heavy isotopes (^15^N, ^18^O) in the residual substrate, a pattern distinct from physical processes like dilution, which reduce concentration without altering isotopic ratios [48,49]. A similar isotopic fractionation pattern is expected to propagate through the biotic reduction of intermediate nitrogen species. However, systematic data on dual N–O isotope dynamics during the reduction of intermediates such as NO_2_^−^ remain scarce [50]. Moreover, recent isotopic studies of abiotic NO_2_^−^ reduction by Fe(II) have revealed fractionation patterns strikingly similar to those expected from biotic pathways [51,52]. As a result, the extent to which isotopic characterization can effectively distinguish between biotic and abiotic nitrite reduction remains unclear. Further research is therefore warranted to refine the application of isotopic tools for elucidating the processes that control nitrate transformation in natural systems [26].

This finding has practical implications for environmental analysis. The available iron content in major soil types typically ranges from several to hundreds of mg·kg^−1^ [53,54,55]. Standard protocols for determining soil nitrate-nitrogen involve an extraction step that brings both nitrate and this available iron into solution. Under suitable acidic and anoxic conditions, the reaction identified in this study could therefore lead to a significant underestimation of nitrate concentration—a possibility also noted by Shao Yanqiu [44]. Further experimental studies are warranted to quantify this potential analytical interference in standard soil testing methods.

## 4. Materials and Methods

### 4.1. Experimental Design

A series of batch experiments were conducted to investigate the effects of molar ratio, reaction time, temperature, and initial pH on the Fe^2+^-mediated reduction of nitrate. Unless otherwise specified, the initial NO_3_^−^ concentration was maintained at 200 mg L^−1^ (as N), all experiments were performed with four replicates, and both nitrate concentration and pH were measured as primary response variables.

#### 4.1.1. Effect of Molar Ratio (n(Fe^2+^):n(NO_3_^−^))

Solutions were prepared with n(Fe^2+^):n(NO_3_^−^) molar ratios of 0.5:1, 1:1, 2:1, 5:1, 10:1, and 20:1. The solutions were mixed at room temperature and analyzed immediately.

#### 4.1.2. Effect of Reaction Time

Solutions were mixed at the selected substrate ratio at room temperature. Nitrate concentration and pH were measured at time points of 0, 10, 20, 30, 40, 50, and 60 min after mixing.

#### 4.1.3. Effect of Reaction Temperature

Solutions were mixed at the selected substrate ratio and then incubated at 0 °C, 15 °C, and 25 °C for designated times. The distilled water used for preparing all solutions was pre-equilibrated in the respective incubators for over 6 h.

#### 4.1.4. Effect of Initial pH

The pH of the NO_3_^−^ solution was adjusted to target values of 4.6, 5.6, and 6.6 using 0.1 mol L^−1^ HCl or NaOH. The solutions were then incubated at 25 °C for the corresponding reaction time. The HCl solution, NaOH solution and distilled water used for preparing all solutions was pre-equilibrated in the respective incubators for over 6 h.

### 4.2. N_2_O Analysis

Nitrous oxide (N_2_O) production was quantified using a Gasera One photoacoustic multi-gas analyzer (Gasera Ltd., Turku, Finland). This instrument employs laser-based photoacoustic spectroscopy, with a detection limit of 50 ug L^−1^) for N_2_O and an average measurement interval of 1 min.

#### 4.2.1. Measurement Under Ambient Air Background

Experiments were conducted in 1 L negative-pressure buffer bottles equipped with two switch valves. Each bottle was filled with 100 mL of the 200 mg L^−1^ NO_3_^−^ solution and 100 mL of the corresponding Fe^2+^ solution. Two experimental setups were used: (1) valves open during the reaction to allow gas exchange with the atmosphere, and (2) valves closed during the reaction to contain gaseous products. After the reaction, the headspace gas was analyzed. The analyzer measured ambient air background for over one hour prior to sample analysis, and for 5 min immediately before each sample measurement. Ambient air (CK) was used as a control.

#### 4.2.2. Measurement Under N_2_ Background

To establish anoxic conditions, experiments were conducted in 1 L negative-pressure buffer bottles equipped with three switch valves. The NO_3_^−^ solution (100 mL) was purged with N_2_ for 10 min to remove dissolved O_2_. The Fe^2+^ solution was prepared using N_2_-purged distilled water and added via syringe. The bottle headspace was purged with N_2_ for approximately 2 min before all valves were sealed. After the reaction, the headspace N_2_O concentration was measured by connecting the bottle to the analyzer in a closed-loop circulation. The N_2_ background was measured for over one hour prior to sample analysis.

### 4.3. Spectroscopic Analysis and Modeling

The characteristic nitrate absorption peak in the 1500–1200 cm^−1^ range of the FTIR-ATR spectra was often obscured by strong water interference, necessitating spectral preprocessing to extract meaningful information. Raw FTIR-ATR spectra were preprocessed using a Savitzky–Golay smoothing filter and baseline correction to reduce noise and correct for drift [56]. The absorption contribution of water was subtracted using a reference band in the 1500–2200 cm^−1^ range [43]. Following preprocessing, the relationship between the nitrate peak intensity and concentration was analyzed using Partial Least Squares Regression (PLSR).

PLSR was employed as the primary chemometric modeling technique. The optimal number of latent variables was determined by minimizing the root-mean-square error (RMSE) of cross-validation using a leave-one-out procedure. Model performance was evaluated on a validation set using the following metrics [57]:

(6) $$\mathrm{RMSE}=\sqrt{\frac{1}{n}\sum_{i=1}^{n}{\left(y_{i}-\hat{y_{i}}\right)}^{2}}$$

(7) $$\mathrm{RPD}=\frac{SD}{RMSE}$$

(8) $$R^{2}=1-\frac{\sum_{i=1}^{n}{\left(y_{i}-\hat{y_{i}}\right)}^{2}}{\sum_{i=1}^{n}{\left(y_{i}-\bar{y}\right)}^{2}}$$

where y_i_ and ŷ_i_ are the measured and predicted nitrate levels for the i-th sample, respectively, ȳ is the mean of measured values, and n is the number of samples. Model robustness was assessed based on high R^2^ and RPD values and a low RMSE. Predictive capability was interpreted using established RPD thresholds: <1.4 (poor); 1.4–1.8 (fair); 1.8–2.0 (good); 2.0–2.5 (very good); and ≥2.5 (excellent) [58].

### 4.4. Chemical Analysis

Solution pH was measured using a standard pH meter [59].

### 4.5. Software

Data analysis was performed using MATLAB R2018a (The MathWorks, Inc., Natick, MA, USA). Graphs were generated using Origin 2024 (OriginLab Corporation, Northampton, MA, USA).

## 5. Conclusions

This study systematically identified the key parameters controlling Fe^2+^-mediated nitrate reduction in an aqueous system. The reaction was rapid within the first 5 min. The Fe^2+^ to NO_3_^−^ molar ratio had a limited, secondary influence on efficiency. Process efficiency exhibited a distinct non-monotonic dependence on temperature, optimal at 25 °C but suppressed at 0 °C and 30 °C. Crucially, initial pH was the predominant governing factor, with a strong negative correlation between final solution pH and nitrate reduction efficiency. A lower initial pH (e.g., 4.6) was essential for creating the acidic environment required for high efficiency. Furthermore, this study provides conclusive evidence that this process is a significant source of the potent greenhouse gas N_2_O under anoxic conditions. In summary, Fe^2+^-driven nitrate reduction is a rapid process governed predominantly by initial pH and finely modulated by temperature. These findings affirm the environmental relevance of this reaction, highlighting its dual role as a potential pathway for nitrogen loss and N_2_O emissions in Fe^2+^- and NO_3_^−^-rich acidic environments and as a possible source of analytical interference in standard nitrate determinations.

## Data Availability

Dataset available on request from the authors.

## Abbreviations

The following abbreviations are used in this manuscript:

DIRB	Dissimilatory Iron-Reducing Bacteria
FTIR-ATR	Fourier-Transform Infrared Attenuated Total Reflectance Spectroscopy
N_2_O	Nitrous Oxide
NRFO	Nitrate-Reducing Fe^2+^ Oxidation
PLSR	Partial Least Squares Regression
R^2^	Coefficient of Determination
RMSE	Root-Mean-Square Error
RPD	Ratio of Performance to Deviation
